# Comparison of Perinatal, Newborn, and Audiometry Results of COVID-19 Pregnant Women

**DOI:** 10.1155/2022/2699532

**Published:** 2022-09-22

**Authors:** Mehmet Yilmaz, Şerif Aksin, Deniz Balsak, Fazil Avci, Osman Özdoğru, Bekir Helvacıoğlu, Mahmut Erdemoğlu, Yasemin Aboalhasan, Gülsüm Doğan

**Affiliations:** ^1^Siirt University, Medical Faculty, Obstetrics and Gynecology, Siirt, Turkey; ^2^Akşehir State Hospital, Obstetrics and Gynecology, Konya, Turkey; ^3^Siirt Training and Research Hospital, Siirt, Turkey

## Abstract

**Objective:**

There are studies on the perinatal outcomes of COVID-19, but the audiometric effects of the maternal immune system against COVID-19 in the newborn are not clear. In this study, we aimed to investigate the relationship between the perinatal outcomes of COVID-19 positive pregnant women and the audiological outcomes of newborns.

**Materials and Methods:**

This retrospective, single-center cohort study was conducted with 65 polymerase chain reaction (PCR) positive pregnant women and newborns and 66 normal pregnant women and newborns who were admitted between January 2020–December 2021. Pregnancy data, perinatal outcomes, and newborn hearing test results of pregnant women and newborns were recorded and compared.

**Results:**

A total of 131 patients were enrolled in the study. The number of normal pregnant women was 66 (50.4%) and the number of pregnant women who had COVID-19 disease was 65 (49.6%). In general, gestational week, age, parity, biochemical parameters, duration of hospital stay, week of delivery, fetal weight, and apgar scores were compared between pregnant women with COVID-19 and normal. White blood cell (WBC), neutrophil, aspartate aminotransferase (AST), and C-reactive protein (CRP) parameters were found to be significantly higher, and lymphocyte and neutrophil/lymphocyte (N/L) ratios were significantly lower (*p* < 0.05). There was no statistically significant difference between the two groups (*χ*^2^=0.001; *p* = 1,000). The normal delivery status, the normal delivery rate in patients with COVID-19 was found to be statistically significantly higher than the cesarean section delivery status (*p* = 0.012). In the statistical comparison between the COVID-19 and normal pregnant groups in the cesarean section group, the gestational week, delivery week, and apgar1 scores of the pregnant women with COVID-19 were found to be significantly higher. There was no statistically significant difference between the distributions of the rate of infants with hearing impairment in the comparison with hearing tests in pregnant women with COVID-19 (*n*=1) and normal pregnant women (*n*=1) (*χ*^2^=0.001; *p* = 1,000).

**Conclusion:**

Although the negative effects of COVID-19 on pregnancy outcomes are rare, it was determined that there was no increased audiological risk factor, and the most important predictor of COVID-19 was lymphopenia.

## 1. Introduction

The coronavirus disease 2019 (COVID-19) pandemic is among the greatest pandemics in human history [1]. Severe acute respiratory syndrome coronavirus 2 (SARS-CoV-2), killed almost 6.2 million people and infected reported over 512 million worldwide as of April 2022 [2]. A successful pregnancy requires well-coordinated communication between mother and fetus. Immune cells and cytokine signaling pathways are mediators of these communications and promote a healthy pregnancy. Certain simultaneous infections or inflammatory conditions in pregnant women cause severe diseases which have detrimental effects on the fetus [3].

The immune response of the placenta and its tropism for specific pathogens and viruses affect the outcome of the pregnant woman's susceptibility to and severity of certain infectious diseases. Generalization of pregnancy as an immune-suppressed condition is misleading and prevents the determination of guidelines for treating pregnant women during pandemics [4, 5]. The suppressed immune system of pregnant women may increase the risk of developing critical or severe diseases associated with COVID-19, in particular pneumonia and respiratory failure [6].

In the meta-analysis study of Jafari et al. [7], it was determined that pregnant and non-pregnant women show the same clinical picture. The case fatality rate of non-pregnant hospitalized patients was 6.4%, and the mortality due to all-cause for pregnant patients was 11.3%. Regarding the complications of pregnancy, postpartum hemorrhage, cesarean delivery, preterm labor, and preterm birth were the most prevalent complications. The rate of vertical transmission was 5.3% (1.3–16), and the rate of positive SARS-CoV-2 tests for neonates born to mothers with COVID-19 was 8% [4–16]. A low risk of vertical transmission is present, and SARS-CoV-2 can be detected in all conception products, particularly placenta and breast milk.

Case reports after COVID-19 infection show that placental and neonatal infection can occur, and maternal infection is associated with placental changes [7, 8]. Therefore, it can be thought that the proinflammatory state of SARS-CoV-2 infections during pregnancy may lead to negative consequences in children [9, 10].

The inner ear is a complex structure that begins to develop in early pregnancy (Carnegie stages 14–16) and progresses during early pregnancy. The third month is the critical period for ear development. Later, the fully developed inner ear may be vulnerable to infections or ototoxic insults [11]. SARS-CoV-2 may indirectly lead to adverse perinatal and long-term neurodevelopmental outcomes [10, 12]. The virus has also been reported to cause sensorineural hearing loss [13, 14]. A COVID-19 infection can cause a wide spectrum of symptoms. The audio-vestibular system can also be involved, but there is still debate about this, so findings need to be considered carefully. Few studies are available about the audio-vestibular symptomatology of newborns with intrauterine COVID-19 exposure [15].

Early detection and intervention for congenital hearing loss are critical for speech and language development. Newborns should receive hearing screening, diagnosis, and intervention at 1, 3, and 6 months, respectively [16]. The COVID-19 pandemic has caused delays in each step of this process. Increased out-of-hospital births and shortages of essential health care services likely reduced the proportion of newborns completing screening [17, 18].

Additional factors have contributed to delayed diagnosis [19].

In our study, we aimed to retrospectively compare the perinatal outcomes of COVID-19 positive pregnant women and the audiological outcomes of newborns.

## 2. Materials and Methods

This retrospective, single-center cohort study included 65 polymerase chain reaction (PCR) positive pregnant women and newborns and 66 normal pregnant women and newborns who were admitted (between January 2020 and December 2021) to the gynecology and obstetrics clinic of Siirt University Training and Research Hospital, which is a tertiary referral center with approximately 3500 deliveries per year. The study was approved by the institutional ethics committee with the approval number 2021/11.01.02. The study was performed according to the standards of the declaration of Helsinki.

### 2.1. Patient Selection

Pregnant women who were diagnosed with coronavirus disease 2019 (COVID-19) infection in the hospital, confirmed by a molecular PCR test performed on a nasopharyngeal swab during pregnancy and at the time of delivery, and all newborns of these pregnant women were included in this study.

The history of COVID-19 infection and the information of all pregnant women who gave birth and their newborns were collected retrospectively from patient medical files from hospital databases. The hearing screening test was applied to every newborn within the scope of the national health system, and the data were collected in an online database that includes mother, newborn, and birth information and can be accessed with a personal identification number. Therefore, even if patients give birth in other hospitals, hearing screening results can be accessed through the system.

Compared parameters of pregnant women with COVID-19 and normal pregnant women were age, gestational week, parity, hemogram, biochemical parameters, length of hospital stay, delivery week, delivery type, fetal weight, Apgar scores, and newborn hearing test results (Auditory brain stem response, ABR test). The hearing screening ABR test of newborns is performed in accordance with the standards of the National Newborn Hearing Screening Program in the hospital.

Inclusion criteria were pregnant women diagnosed with COVID-19 infection in the hospital, confirmed by molecular PCR tests performed on nasopharyngeal swabs during pregnancy and delivery, and hearing tests of all newborns and newborn babies of these pregnant women. Exclusion criteria from the study were accepted as the history of TORCH infection in the mother, familial hearing impairment, head and neck anomalies related to the external auditory canal and middle ear, <1500 g birth weight, birth with a low apgar score, neonatal hyperbilirubinemia, bacterial meningitis, ototoxic drug use and long-term mechanical intubation, miscarriage with apgar scores, and newborns with syndromes.

### 2.2. Hearing Test Application

A neonatal hearing test was performed in the first two weeks after birth using the ABR test. If a newborn failed the ABR test, a second ABR test (ABR reference) was performed two weeks later. Newborns were evaluated bilaterally while sleeping without sedation. All newborns underwent otoscopic examination by a specialist before the ABR test. Newborns who failed the second ABR test were referred to the secondary otolaryngology center. In the ABR test, the auditory thresholds were determined as normal if ≤20 dB and hypoacusis (unilateral or bilateral) if ≥20 dB (mild 21–40 dB, mean 41–70 dB, severe 71–90 dB, deep >90 dB). In the case of hypoacusis, the child was invited to perform a new ABR test at 6 months of age to confirm or exclude a diagnosis of sensorineural hearing loss. Mothers and newborns in the control group were selected from the hospital database with dates of birth matching the study group, among pregnant women of the same age and parity.

### 2.3. Statistical Methods

Statistical analyzes were performed with SPSS version 26.0 software. The conformity of the variables to the normal distribution was examined with the Kolmogorov–Smirnov/Shapiro–Wilk tests. In the analysis of the data, *t*-test was used for independent groups in normal distribution and the Mann–Whitney *U* test was used in the case of non-normal distribution. Pearson's chi-Square test was used to compare categorical variables. A *p* value below 0.05 was considered statistically significant.

## 3. Results

131 patients were included in the study. The number of normal pregnant women was 66 (50.4%) and the number of pregnant women who had COVID-19 disease was 65 (49.6%). In general, gestational week, age, parity, biochemical parameters, duration of hospital stay, week of delivery, fetal weight, and Apgar scores were compared between pregnant women with COVID-19 and normal. White blood cell (WBC), neutrophil, aspartate aminotransferase (AST), and C-reactive protein (CRP) parameters were found to be significantly higher, and lymphocyte and neutrophil/lymphocyte (N/L) ratios were significantly lower (*p* < 0.05). The descriptive statistical analysis of the laboratory findings and demographic information of all patients are given in Table 1.

There was no statistically significant difference between the distributions of the rate of infants with hearing impairment in the comparison with hearing tests in pregnant women with COVID-19 (*n*=1) and normal pregnant women (*n*=1) (*χ*^2^0.001; *p*=1,000).

When the delivery method was compared according to the patient's COVID-19 or normal delivery status, the normal delivery rate in patients with COVID-19 was found to be statistically significantly higher than the cesarean section delivery status (*p*=0.012).

A statistically significant difference was found in WBC, lymphocyte, neutrophil, neutrophil/lymphocyte ratio, alanine aminotransferase (ALT), and CRP parameters in the normal delivery group between the COVID-19 and normal pregnant groups. In the group with COVID-19, lymphocyte, and N/L values were significantly lower, and WBC, neutrophil, ALT, and CRP values were significantly higher compared to the normal group. In the statistical comparison between the COVID-19 and normal pregnant groups in the cesarean section group, the gestational week, delivery week, and Apgar1 score of the pregnant women with COVID-19 were found to be significantly higher(Table 2).

## 4. Discussions

Studies were carried out on the effects of COVID-19 on pregnancy, pregnancy outcomes, and newborns. Available evidence indicates that COVID-19 infection during pregnancy rarely affects fetal and neonatal mortality [20]. However, COVID-19 can affect multiple organs and systems [21, 22]. It mainly affects the respiratory system and can cause a wide range of symptoms in the respiratory system—from the common cold to severe respiratory distress [23]. Fetuses may be exposed to SARS-CoV-2 during critical periods of fetal development [24] because the immunopathogenesis of the disease is not completely clear [25].

The COVID-19 pandemic jeopardizes each of the steps required for EHDI (early detection and early intervention) [16]. Normally, most babies are screened for hearing at one month of age before being discharged from the maternity hospital. However, studies show that there is an increase in out-of-hospital deliveries during the pandemic [26–28], and therefore, babies born outside the hospital are more likely to delay or fail in terms of full newborn hearing screening [29]. However, in this study, due to the regular screening applied by the Ministry of Health, no difference was found between pregnant women with COVID-19 and those who did not, without delay.

A total of 984 neonates were included (508 males and 476 females) in a multicenter study by Mostafa et al. [13] to determine the possible effect of maternal SARS-COV-2 infection on neonatal hearing as identified during universal hearing screening. Neonates born to Covid-19 positive mothers do not seem to have an increased risk of hearing loss. Likewise, in this study, no increased hearing loss was detected in babies born to COVID-19 positive mothers.

Yoon et al. found that it was associated with adverse pregnancy outcomes such as preterm birth and low birth weight in their systematic review of 201 newborns born to 223 pregnancies with COVID-19 [30]. Likewise, in the meta-analysis of 42 studies involving 438.548 pregnant women, Shu et al. found that the risk of preterm birth and low birth weight increased in pregnant women with COVID-19 [31]. Abdelazim et al. reported that COVID-19 increased the risk of premature birth. Karaçam et al. expressed the risk of premature birth as 18% [32, 33].

In our study, there was no statistical difference in terms of the week of birth and fetal weight between pregnant women with and without COVID-19 . The study of Shu et al. differs from this study in terms of its results, as it is both a meta-analysis study and a large number of cases.

Oskovi-Kaplan et al. [14] investigated the incidence of the risk of neonatal hearing loss in infants of mothers who had COVID-19 infection during pregnancy, regarding their trimesters, by evaluating the neonatal hearing screening results. In the retrospective case-control study, neonatal hearing test results of 458 women with a history of COVID-19 infection in pregnancy were compared with those of 339 women who gave birth before the pandemic. Neonates born before 34 weeks, and with reported risk factors in the database such as congenital anomaly or known TORCH infection during pregnancy were excluded. The screening tests, automated auditory brainstem response or transient evoked otoacoustic emission (TEOAE), were used for screening, and patients who failed the first screening were reevaluated at least 2 weeks apart with a second screening. COVID-19 infection during pregnancy was not found to be a risk factor for hearing loss, according to the newborn hearing screening results. Cesarean section rate was found to be higher in Covid pregnant women compared to the control group (56.2% vs. 32.6%), but the week of delivery and birth weight were found to be similar. In this study, the normal birth rate was found to be higher, but it is similar in terms of other parameters. It may be due to the difference between centers in terms of delivery method and the absence of cesarean section indications in the studies.

Ghiselli et al. [15] enrolled 63 children born to mothers who had contracted COVID-19 during pregnancy, and investigated the possible correlation between the COVID-19 gestational infection and hearing impairment onset in newborns. Children were subjected to newborn hearing screening and audiological evaluation. Newborn hearing screening is carried out prior to hospital discharge using the automatic transient evoked otoacoustic emissions test. An audiological evaluation is performed at the baby's age of 4 months by using maternal pregnancy and perinatal case history; COVID-19 case history; otoscopy; acoustic immittance test; distortion product otoacoustic emissions test and the auditory brainstem response test. The study found no evidence that maternal COVID-19 infection is a risk factor in the development of congenital hearing loss in newborns. Advanced maternal age (32 years), the inclusion of the TORCH infection panel, the presence of drug use during pregnancy, better apgar scores at 1st and 5th minutes (9.2–9.9, respectively), and the absence of a control group are the differences from this study. Although COVID-19 has drug use and accompanying diseases, its results are compatible with this study.

In the meta-analysis study of Jafari et al. [34], it was determined that pregnant and non-pregnant women showed the same clinical picture. Pregnant women have a higher proportion of leukocytosis (27% vs. 14%) and thrombocytopenia (18% vs. 12%) and a lower proportion of raised C-reactive protein (52% vs. 81%) compared with nonpregnant patients. Leucopenia and lymphopenia were almost the same in both groups. Higher odds of cesarean delivery, low birth weight, and preterm birth among pregnant patients with COVID-19 suggest a possible association between COVID-19 infection and pregnancy complications. While the compatible part with this study is leukocytosis, leukocytosis, and lymphopenia, differences in our study are the groups consisting of pregnant women with and without COVID-19, high CRP level in COVID-19 pregnant women, and low Neutrophil/Lymphocyte ratio. However, while there was no statistical difference in terms of the week of birth and fetal weight between pregnant women with and without COVID-19, contrary to this meta-analysis, the rate of CS was found to be higher in pregnant women without COVID-19. This meta-analysis study consists of pregnant and non-pregnant groups, and thrombocytopenia and CRP levels were found to be lower in pregnant women compared to non-pregnant women. In addition, an increased CS rate, low birth rate, and preterm birth risk were found in COVID-19 pregnant women. [34].

Dey et al. reported that the rate of cesarean section is higher in pregnant women with Covid than in normal pregnancy [35]. On the contrary, there are studies that do not provide enough evidence about the relationship between COVID-19 and maternal-fetal and perinatal complications [36].

The difference in cesarean section rates between studies may be due to different indications and sociodemographic characteristics. In addition, studies may be from heterogeneous groups, concomitant diseases, and clinical pictures.

In a meta-analysis study of 364 pregnant women and 302 newborns with a diagnosis of COVID-19, Mona et al. [37] showed that the course of COVID-19 in pregnant women was similar to pregnancy outcomes in other populations. Similar results were also reported in the study of Maryam et al. [20]. In addition, laboratory values of hb, platelet, AST, ALT, Cr, and BUN were within normal limits, while increased CRP neutrophilia and lymphopenia were detected. Increased crp neutrophilia and lymphopenia are the most common laboratory results in Vakili et al., Nikpour et al., and other studies [38–40]. Although lymphopenia is physiologically normal during pregnancy, a sharp decrease in lymphocytes is used as a good predictor of COVID-19-related mortality [41]. Our study is in agreement with data from two studies.

The limitations of our study are that it is retrospective, single-centered, not randomized, the number of cases is small, and the severity of COVID-19 is not defined, while its strengths are its comparison with the control group, detailed proinflammatory parameters, and regular case records.

Healthcare services opportunities exist to continue to reduce the negative impact of COVID-19 on neonatal hearing health care. It is recommended that training for a hearing test, registration, and follow-up of newborns be repeated regularly. Incomplete follow-up should be referred to pediatric audiology immediately. The follow-up and rehabilitation of children with hearing loss affected by COVID-19 will continue to be important in the coming years [18].

In summary, although the negative effects of COVID-19 on pregnancy outcomes are rare, it has been shown that it is not an audiological risk factor. The most important predictive parameter of COVID-19 was found to be lymphopenia. Randomized controlled studies are needed for the exact reason why it is an audiological risk factor.

## Figures and Tables

**Table 1:** Comparison of biochemical variables between COVID and normal pregnant groups.

Variables	Pregnant groups		*p* value
	COVID-19	Normal	
Gestational week	38.9 ± 1.7	38.9 ± 1.1	0.995
Age, year	26.7 ± 4.7	26.9 ± 4.7	0.777
Parity, n	2.8 ± 1.6	2.9 ± 1.6	0.735
WBC, K/uL	16.3 ± 5.5	13.6 ± 4.2	**0.002**
Lymphocyte, K/uL	10.7 ± 6.1	13.1 ± 7.1	**0.046**
Neutrophil, K/uL	82.3 ± 6.5	79.6 ± 8.4	**0.043**
Neutrophil/Lymphocyte ratio	0.14 ± 0.1	0.18 ± 0.1	**0.043**
Hemoglobin, g/dL	11.25 ± 1.5	11.2 ± 1.4	0.846
Htc, %	35.3 ± 4.2	35.0 ± 3.4	0.646
Platelet, K/uL	234.5 ± 61.1	233.2 ± 66.8	0.905
Glucose, mg/dL	91.2 ± 25.8	92.6 ± 23.3	0.752
Urea, mg/dL	18.5 ± 5.1	17.3 ± 5.0	0.209
Creatinine, mg/dL	0.6 ± 0.1	0.59 ± 0.1	0.375
AST, U/L	28.0 ± 9.5	24.8 ± 6.4	**0.025**
ALT^*∗*^, U/L	18.0 ± 12.2	14.5 ± 6.1	0.042
D-dimer, ug/L	971.3 ± 647.7	781.0	NA
CRP^*∗*^, mg/dL	14.9 ± 21.6	7.8 ± 15.1	**0.031**
Ferritin^*∗*^, ng/mL	28.4 ± 19.8	21.1 ± 19.7	0.230
Duration of hospital stay	2.5 ± 0.9	2.5 ± 0.8	0.758
Week of birth	38.9 ± .1.7	38.9 ± 1.1	0.941
Fetal weight, gr	3214.5 ± 542.7	3248.5 ± 375.0	0.679
Apgar1. min	7.8 ± 1.1	7.9 ± 0.6	0.741
Apgar 5. min	9.0 ± 1.2	9.1 ± 0.5	0.447
ARB test	1	1	1.000

AST, aspartate aminotransferaz; ALT, alanine aminotransferaz; CRP: C-reactive protein. Independent t test and ^*∗*^Mann–Whitney U test were used and *p* < 0.05 was considered significant. NA; not applicable.

**Table 2:** Comparison of biochemical variables between COVID-19 and normal pregnant groups in normal and cesarean delivery.

Variables	Normal delivery groups		*p* value	Cesarean delivery groups		*p* value^*∗*^
	COVID-19 (*n*=57)	Normal (n=46)		COVID-19 (n=8)	Normal (n=20)	
	Mean ± SD/median (IQR)	Mean ± SD/median (IQR)		Median (IQR)	Median (IQR)	
Gestational week	38.8 ± 1.8	39.2 ± 0.9	0.185	39 (1)	38.5 (1)	0.117
Age, year	26.8 ± 4.8	27.2 ± 4.6	0.634	25.5 (8)	26 (6)	0.838
Parity, *n*	2.9 ± 1.6	3.1 ± 1.5	0.506	2 (3)	2 (2)	0.812
WBC, K/uL	16.4 ± 5.6	13.8 ± 4.5	**0.012**	15.5 (9)	12 (5)	0.185
Lymphocyte, K/uL	10.3 ± 6.2	14.0 ± 7.8	**0.010**	13.5 (9)	11 (7)	0.160
Neutrophil, K/uL	82.6 ± 6.7	78.4 ± 8.8	**0.008**	80 (6)	84 (10)	0.169
Neutrophil/lymphocyte ratio	0.13 ± 0.1	0.19 ± 0.14	**0.013**	0.17 (0.12)	0.13 (0.10)	0.186
Hemoglobin, g/dL	11.3 ± 1.4	11.3 ± 1.4	0.895	12 (3)	11 (1)	0.711
Htc, %	35.5 ± 4.1	35.2 ± 3.6	0.722	35.5 (9)	35 (4)	0.939
Platelet, K/uL	234.3 ± 61.2	241.2 ± 66.0	0.589	227.5 (117)	214.5 (86)	0.509
Glucose, mg/dL	91.5 ± 26.9	95.9 ± 25.8	0.401	88 (34)	86 (22)	0.610
Urea, mg/dL	18.3 ± 5.0	17.9 ± 5.2	0.686	18.5 (7)	16 (4)	0.071
Creatinine, mg/dL	0.6 ± 0.1	0.59 ± 0.1	0.660	0.6 (0.1)	0.6 (0.1)	0.226
AST, U/L	28.4 ± 9.7	25.3 ± 6.9	0.071	26 (16)	24 (6)	0.557
ALT^*∗*^, U/L	15 (7)	12.5 (7)	**0.037**	13.5 (9)	15 (6)	0.523
D-dimer, ug/L	1051.3 ± 675.2	−(n=0)	NA	−(*n*=2)	−(*n*=1)	NA
CRP^*∗*^, mg/dL	8 (11)	4 (4)	**0.008**	13 (22)	6 (5.5)	0.052
Ferritin^*∗*^, ng/mL	22 (34) (*n*=23)	11 (44) (*n*=11)	0.699	14 ()	10.5 (13.5)	0.410
Duration of hospital stay, day	2.5 ± 0.9	2.2 ± 0.8	0.152	3 (1)	3 (0)	0.252
Week of birth	38.8 ± .1.8	39.2 ± 0.9	0.140	40 (1)	38.5 (1)	**0.004**
Fetal weight, gr	3187.5 ± 531.8	3237.2 ± 377.0	0.623	3130 (1176)	3220 (479)	0.958
Apgar1. min	7.7 ± 1.2	8.0 ± 0.5	0.235	8 (1)	7.5 (1)	**0.013**
Apgar 5.min	8.9 ± 1.3	9.1 ± 0.5	0.232	9 (1)	9 (0)	0.059

AST, aspartate aminotransferase; ALT, alanine aminotransferase; CRP, C-reactive protein, white blood cell (WBC); N/L, neutrophil/lymphocyte ratio, NA, not applicable. Independent *t* test and ^*∗*^Mann–Whitney *U* test were used and *p* < 0.05 was considered significant.

## Data Availability

Data are available on request.
